# Supplementary material for: A suicide safety protocol with vulnerable populations with chronic physical health conditions: A pragmatic protocol implementation among displaced Myanmar adults in Thailand
Source: Glob Ment Health (Camb). 2026 Feb 19;13:e35. doi: 10.1017/gmh.2025.10101 (PMC12951338; doi:10.1017/gmh.2025.10101)
Supplement: Sardana et al. supplementary material [file S2054425125101015sup001.docx]

### **Key Messages**

- Suicide safety protocols are a necessary and feasible aspect of psychosocial support programs.
- Implementing evidence-based suicide screening and safety management techniques within routine mental health interventions for chronic disease patients can facilitate early detection of suicide risk and make management more efficient as part of routine care that clients receive.
- Consideration of particularly vulnerable populations (such as ethnic minorities, older adults, clients with physical disabilities and limited mobility) may require additional strategies to ensure to adoption of suicide risk reduction strategies included in the protocol.
- Safety protocol implementation adaptation is an iterative process throughout the duration of a program execution cycle requiring continuous and consistent collaboration with local staff.
